# The Nature of Triel Bonds, a Case of B and Al Centres Bonded with Electron Rich Sites

**DOI:** 10.3390/molecules25112703

**Published:** 2020-06-11

**Authors:** Sławomir J. Grabowski

**Affiliations:** 1 Kimika Fakultatea, Euskal Herriko Unibertsitatea UPV/EHU, and Donostia International Physics Center (DIPC), P.K. 1072, 20080 San Sebastian, Spain; s.grabowski@ikerbasque.org; Tel.: +34-943-018-187; 2 IKERBASQUE, Basque Foundation for Science, 48011 Bilbao, Spain

**Keywords:** triel bond, boron centre, aluminium centre, tetrahedral structure, quantum theory of atoms in molecules, natural bond orbital method, π-hole bond

## Abstract

The second-order Møller–Plesset perturbation theory calculations with the aug-cc-pVTZ basis set were performed on complexes of triel species: BCl_3_, BH_3_, AlCl_3_, and AlH_3_ acting as Lewis acids through the B or Al centre with Lewis base units: NCH, N_2_, NH_3_, and Cl^−^ anion. These complexes are linked by triel bonds: B/Al⋅⋅⋅N or B/Al⋅⋅⋅Cl. The Quantum Theory of ´Atoms in Molecules´ approach, Natural Bond Orbital method, and the decomposition of energy of interaction were applied to characterise the latter links. The majority of complexes are connected through strong interactions possessing features of covalent bonds and characterised by short intermolecular distances, often below 2 Å. The BCl_3_⋅⋅⋅N_2_ complex is linked by a weak interaction corresponding to the B⋅⋅⋅N distance of ~3 Å. For the BCl_3_⋅⋅⋅NCH complex, two configurations corresponding to local energetic minima are observed, one characterised by a short B⋅⋅⋅N distance and a strong interaction and another one characterised by a longer B⋅⋅⋅N distance and a weak triel bond. The tetrahedral triel structure is observed for complexes linked by strong triel bonds, while, for complexes connected by weak interactions, the structure is close to the trigonal pyramid, particularly observed for the BCl_3_⋅⋅⋅N_2_ complex.

## 1. Introduction

The number of experimental and theoretical studies concerning inter- and intramolecular interactions increased rapidly in recent years [1,2,3,4,5,6,7]. The concept of σ-holes and π-holes is one of the most important trials to explain nature of the broad spectrum of interactions [2,3]. The aerogen (group 18), halogen (group 17), chalcogen (group 16), pnicogen (group 15), and tetrel (group 14) atoms [1,2,3,4,5,6,7] may act as the Lewis acid centres interacting with electron-rich sites; these interactions taking their names from names of corresponding centres are often classified as σ-hole bonds [2,3]. In the above-mentioned centres, the depletion of the electron charge may be observed on their edges in directions of their bonds with other atoms. These sites are just named as σ-holes; the above-described electron charge outflow is often sufficient to lead to the positive electrostatic potential (EP) at the σ-holes and, thus, to their Lewis acid properties.

It may be confusing that the centres described here play a role of Lewis acids since the majority of them are electronegative atoms. Some of these centres may be characterised by π-holes that are also regions of the depletion of the electron charge and, consequently, often of the positive electrostatic potential. The π-holes occur for planar species or planar molecular fragments in directions perpendicular to the corresponding planes [2,3]. The elements of the 13th group in their numerous compounds are characterised by the occurrence of π-holes. The corresponding interactions named as triel bonds are also classified as π-hole bonds [8,9,10]. The triel bonds usually designate interactions between the triel centre characterised by the positive electrostatic potential, i.e., by the Lewis acid properties, and the electron-rich site, lone electron pair, π-electrons, etc. This term covers the broad spectrum of interactions, from very weak to those of a covalent nature, but most of them are quite strong [8,9,10]. The boron trihydrides and trihalides are examples of simple planar species with the boron centre possessing positive electrostatic potential that may interact with nucleophiles. It was found that the acidity of the boron centre increases in the following order: BF_3_ < BCl_3_ < BBr_3_ < BI_3_ [11], which seems to be surprising since the electronegativity of the halogen substituent decreases in this order. Such increase of acidic properties was explained by the back bonding effect [12], where it is the greatest for the BF_3_ species leading to the significant decrease of the positive charge of boron, i.e., to the decrease of its acidic properties. However, other studies showed that this effect is negligible [13,14]. This led to numerous discussions and polemics concerning the nature of the triel centre interactions [15,16]. It seems that the Lewis base units interacting with these boron trihalides also play a crucial role since it was found that the above-mentioned order depends on the nature of the electron-donating centres [17,18].

Another topic related to boron trihalides interacting with Lewis bases concerns the double potential energy minimum for some of such complexes [19,20,21,22]. In the case of the BF_3_⋅⋅⋅CH_3_CN complex, two configurations corresponding to the energetic minima were found characterised by N⋅⋅⋅B distances amounting to 1.818 Å and ~2.3 Å. The corresponding binding energies for these configurations are equal to −7.7 kcal/mol and −8.7 kcal/mol, respectively [20,21]. For the BCl_3_⋅⋅⋅CH_3_CN complex, two local energetic minima were found that are related to configurations with the N⋅⋅⋅B distances equal to 1.601 Å and 2.687 Å; the binding energies amount to −12.0 and −4.9 kcal/mol, respectively [22]. It seems that it may be a more general problem for boron centres since, in numerous molecular systems, they are obscured by various types of substituents. This may concern other centres since the similar situation of the double potential minimum was observed for complexes of acetonitrile with tetrahalides of elements of the fourth and 14th groups [23]. It was analysed in this study if such double minima occur for the aluminium trichlorides and trihydrides.

The triel bonds discussed in this study were compared with hydrogen bonds recently [24]. It was also discussed that different types of triel bonds are observed, similarly as for the other interactions. This means that the triel centres may interact with single electron-rich sites, as well as with π-electron systems, like acetylene and ethylene and their derivatives. The complexes of simple triel species with benzene were also analysed recently [25]. The intramolecular triel bonds occur in crystal structures. For example, the interactions in hydrides and halides of 1,2-bis(dichloroboryl)benzene and 1,8-bis(dichloroboryl)naphthalene were analysed theoretically, where these systems and similar ones were also found in crystal structures; the corresponding interactions may be classified as intramolecular triel bonds or bifurcated triel bonds [26].

The next matter concerns different properties of different triel elements. It was discussed that characteristics of triel bonds depend on the kind of triel centre in contact with the electron-rich site, i.e., boron, aluminium, gallium, or other 13th group element [8]. These interactions are often very strong, and they possess characteristics of covalent bonds. Cambridge Structural Database [27,28] searches were performed recently, and it was found that the boron centre possesses coordination four most often, in ~64% of all cases, while this coordination for the aluminium centre occurs for ~70% of structures [24]. Coordination three is not so common in both cases in crystal structures, with occurrences of 13.9% and 3.3%, respectively [24]. This is because the trivalent triel centres do not obey the octet rule and, consequently, they are very reactive, strongly interacting with Lewis bases to form tetrahedral structures. The aim of this study is to characterise properties of triel bonds where boron and aluminium centres are in contact with electron-rich sites. The possibility of the existence of the double potential energy minima is also analysed here.

## 2. Results and Discussion

### 2.1. The Strength of Triel Bonds

**Figure 1:**
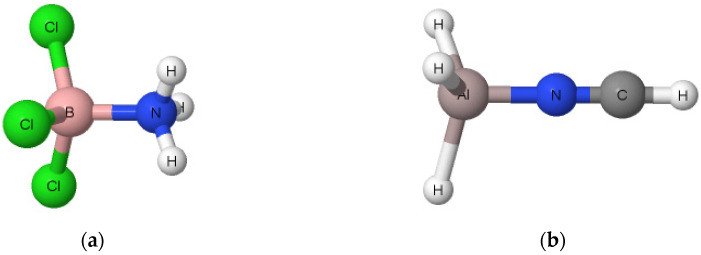
Examples of two complexes analysed in this study: (**a**) BCl_3_⋅⋅⋅NH_3_; (**b**) AlH_3_⋅⋅⋅NCH.

**Scheme 1:**
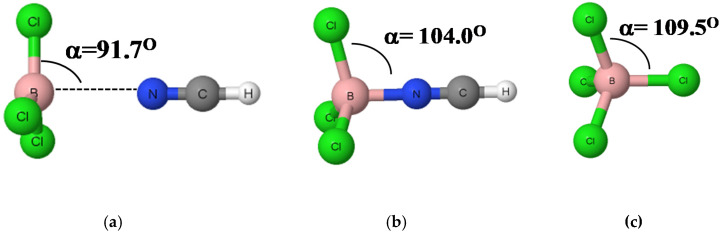
The definition of the α-angle; examples of three species with the corresponding values of α are presented: (**a**) BCl_3_⋅⋅⋅NCH (configuration with the longer B⋅⋅⋅N distance), (**b**) BCl_3_⋅⋅⋅NCH (configuration with the shorter B⋅⋅⋅N distance), and (**c**) BCl_4_^−^ species.

The calculations were performed for complexes of the BCl_3_, BH_3_, AlCl_3_, and AlH_3_ species acting as the Lewis acid units with the following Lewis bases: NCH, N_2_, NH_3_, and Cl^−^ anion. Figure 1 and Scheme 1 present a few examples of systems analysed here.

**Figure 2:**
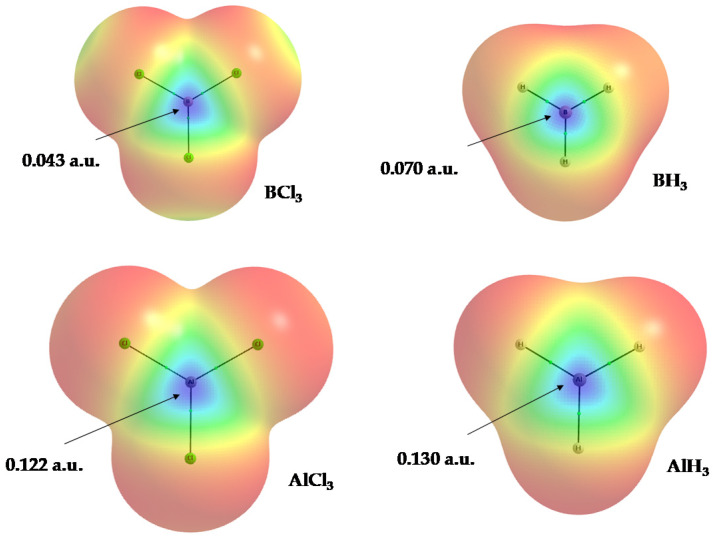
The electrostatic potential maps for molecular surfaces with the electron density of 0.001 au. The following species are presented: BCl_3_ and BH_3_ (up), as well as ACl_3_ and AlH_3_ (below); the colour scale is fixed separately for each of the species presented, from maximum positive values (blue) to minimum negative ones (red).

Figure 2. presents maps of the electrostatic potential (EP) for the molecular surfaces of Lewis acid units characterised by the electron density of 0.001 au. One can see regions of positive EP at the triel centres that correspond to the π-holes; values of the maximum EP are also presented. These positive EPs at π-holes determine the Lewis acid properties of triel centres that may be connected with electron-rich sites. The EP value is greater for trihydride than for the corresponding trichloride; moreover, EP is greater at the aluminium centre than at the boron centre of the corresponding species. The lowest maximum value of EP for the BCl_3_ species may be explained by the influence of chlorine substituents on the boron centre. However, there is no clear dependence of the EP value on the strength of the corresponding triel bond, as results discussed further here show. Thus, this means that not only electrostatic forces determine the strength of these interactions.

The energetic parameters of complexes linked by triel bonds and analysed here are presented in Table 1. The interaction and binding energies, E_int_ and E_bin_ [29], respectively, are included, as well as the deformation energy, E_def_ [30], and the basis set superposition error correction, BSSE [31]. The interaction energy is the difference between the energy of the complex and the sum of energies of interacting units with their geometries taken from the geometry of the complex. One may say that it describes the strength of interaction of the link in complex. In the binding energy, the energies of the interacting units correspond to their geometries optimised separately. In such a way, E_bin_ takes into account the deformation of these units resulting from the complexation, E_bin_ = E_int_ + E_def_. The latter term, the deformation energy, may be understood as “the energetic cost” of the complex formation. There are systems where E_int_ is negative, indicating the local stabilising interaction, while E_bin_ is positive, showing that the formation of the complex is not energetically preferred [32]; this may correspond to an endothermic reaction. This is not a case for complexes analysed here, where both interaction and binding energies are negative for all cases (Table 1).

There is a sub-group of complexes analysed here, with anions being products of interactions between the trivalent triel species and the chloride anion. The large −E_int_ and −E_bin_ values indicate that they are stable systems with interactions corresponding to exothermic reactions. Large deformation energies are observed for these anionic systems. In the BCl_4_^−^ anion, this energy is close to 40 kcal/mol. One can see that the formation of these anions leads to great deformations of geometries of interacting units where the planar trichlorides and trihydrides are transformed into the tetrahedral structures. For example, the BCl_4_^−^ (see Scheme 1) and AlCl_4_^−^ anions are characterised by the Cl–B/Al–Cl angle of 109.5° as for methane and other ideal tetrahedral species. The corresponding H–B/Al–Cl angle for the BH_3_Cl^−^ and AlH_3_Cl^−^ anions is equal to 106.5° and 107.1°, respectively (Table 2). These anions, being the result of interaction of trivalent B and Al species with chloride, are stable tetravalent systems where the triel centre obeys the octet rule.

Table 1 shows that other, neutral complexes are characterised by a broad spectrum of interactions, from −E_int_ amounting to 1.5 kcal/mol for the BCl_3_⋅⋅⋅N_2_ complex, to 50.4 kcal/mol for the BCl_3_⋅⋅⋅NH_3_ one. The deformation energy for neutral complexes is greater for stronger interactions. For the BCl_3_⋅⋅⋅NH_3_ complex, the E_def_ value amounts 23.6 kcal/mol. For the very weak interaction in the BCl_3_⋅⋅⋅N_2_ complex, E_def_ is negligible, equal to 0.02 kcal/mol, while, for another weak interaction in the AlH_3_⋅⋅⋅N_2_ complex, it amounts to 0.7 kcal/mol. The complexes linked by weak interactions are marked in all tables by superscript a, while those classified as connected by strong interactions are marked by superscript b. However, there is no sharp border between strong and weak interactions, and the division proposed here is rather contractual. These relationships between the deformation energy and the parameters describing strength of interaction were observed earlier for complexes linked by triel bonds [10,24]. The BSSE correction is located in the range 1–2 kcal/mol for the majority of systems considered here; for a few boron complexes, it is greater than 2 kcal/mol, while, for a few aluminium species, it is lower than 1 kcal/mol.

There are two configurations of the BCl_3_⋅⋅⋅NCH complex, characterised by the energies of interaction amounting to −23.6 kcal/mol and −3.5 kcal/mol with the corresponding B⋅⋅⋅N distances of 1.628 Å and 2.817 Å, respectively (Table 2). However, the binding energies of these two configurations are almost equal since, for that characterised by the shorter B⋅⋅⋅N distance, the complexation is connected with great changes of geometries of interacting units. The Cl–B–N angle amounts here to 104°, and the deformation energy is equal to 19.8 kcal/mol. For the configuration with the longer B⋅⋅⋅N distance, these values are equal to 91.7° and 0.3 kcal/mol, respectively.

The angle mentioned above is designated as α hereinafter; it is between the Cl/H–B/Al bond of the Lewis acid unit and the B/Al⋅⋅⋅N/Cl intermolecular distance (Scheme 1). In the case of strong interactions, complexes are close to the tetrahedron structure, while, in the case of weak interactions, the Lewis acid unit remains planar, or nearly so, and the α angle defined above is equal or close to 90°. For two configurations of the BCl_3_⋅⋅⋅NCH complex, one of them is close to the tetrahedral structure and the other one is close to the trigonal pyramid with the Lewis acid unit close to the planarity.

The α angles are collected in Table 2, while the intermolecular distances are also included there, as well as the selected the Natural Bond Orbital (NBO) [33,34] parameters. If one refers to the consistent van der Waals radii proposed by Truhlar and co-workers (N—1.55 Å, Cl—1.75 Å, B—1.92 Å, Al—1.84 Å) [35], all intermolecular distances are shorter than the corresponding sum of van der Waals radii. However, the term “intermolecular” should be used with caution since, for the tetrahedral anionic species, all links to the triel centre possess characteristics of covalent bonds. The stability of the similar anion, BF_4_^−^, was discussed recently [36]. The number of structures containing the BF_4_^−^ anion amounts to 16,088 according to search performed through the Cambridge Structural Database (CSD updates up to March 2020). The search performed here for the same version of CSD to find BCl_4_^−^ ions shows only 36 crystal structures. However, there are no structures containing BCl_3_ neutral species. It was pointed out that trigonal boron trihalides occur only in the gas phase [37]; it seems they are very reactive possessing strong Lewis acid properties, and they react with nucleophiles in more condensed phases.

The electron charge shift from the Lewis base to the Lewis acid unit that results from complexation calculated within the NBO approach is included in Table 2. One can see that greater electron charge shifts are observed for stronger interactions. The greatest shifts between −0.38 au and −0.69 au occur for anionic complexes, as well as for the BCl_3_⋅⋅⋅NH_3_ and BH_3_⋅⋅⋅NH_3_ complexes, while the smallest ones, lower than −0.01 au, occur for complexes linked by the weakest interactions, such as the BCl_3_⋅⋅⋅N_2_ complex and the BCl_3_⋅⋅⋅NCH configuration characterised by the longer B⋅⋅⋅N distance. Table 2 presents the atomic charge of the Lewis acid centre, boron, and aluminium. For all complexes analysed, their formation leads to this charge decrease, up to negative values for the boron centre in the BH_3_ complexes. There are only two exceptions to the above rule; for the weakest interactions in the BCl_3_⋅⋅⋅N_2_ complex and in one of the BCl_3_⋅⋅⋅NCH configurations, the complexation leads to the increase of the positive charge of the boron centre. The Lewis acid properties of the triel centres are supported by the positive electrostatic potentials (EPs) at their surfaces that are related to the above-mentioned π-holes; the positive EPs for simple species of boron and other triel elements were analysed in former studies in detail [8,9,10,38].

It is worth recalling that, for the latter two cases of weakest interactions, the smallest α-angles occur; thus, the BCl_3_ part of the complex is close to the planarity. For all remaining complexes, the NBO approach detects the σ-bond orbital between the boron or aluminium centre and the nitrogen or chlorine Lewis base site. The polarisation of the latter link is shown in Table 2. It is understood as the percentage of the electron density at the triel (B or Al) centre. One can see that there is no clear tendency here. The greatest concentrations of the electron density at the triel centre occur for the AlCl_3_ complexes, as well as for the AlH_3_⋅⋅⋅N_2_ and AlH_3_⋅⋅⋅NCH complexes, above 90% (except of the AlCl_4_^−^ complex where it amounts to 83.5%). In the case of AlH_3_⋅⋅⋅NH_3_ and AlH_3_Cl^−^, the polarisation is equal to 8.5% and 15.1%, respectively, which means that the electron density for the Al–N and Al–Cl links, respectively, is accumulated mainly at nitrogen and chlorine centres. The analysis of these intermolecular links that may be classified as triel bonds, based on results collected in Table 1 and Table 2, shows that their properties for aluminium and boron centres differ.

### 2.2. Double Minima for Triel–Lewis Base Potential Energy Curves

It was mentioned earlier here that there are examples of complexes of simple boron species that are characterised by two configurations corresponding to energetic minima, i.e., by the double minimum of potential energy [19,20,21,22,38]. This also concerns simple moieties of elements of the fourth and 14th groups [23]. In the case of triel species, two configurations were discussed for the BCl_3_⋅⋅⋅CH_3_CN and BF_3_⋅⋅⋅CH_3_CN complexes; however, in the latter case, one of the potential energy minima is not well separated [20,21,22]. Two configurations were shortly discussed recently for the BCl_3_⋅⋅⋅NCH complex, and two local energy minima were also found for the BBr_3_⋅⋅⋅NCH and BI_3_⋅⋅⋅NCH complexes [38]. It was concluded that the existence of two configurations in simple triel species is the result of the balance of interaction energy terms, and that Pauli repulsion plays a crucial role [19]. In a more recent study, it was discussed that the double minimum results from the balance of electrostatic interactions between the BX_3_ (X = Cl, Br, I) and HCN units [38].

**Figure 3:**
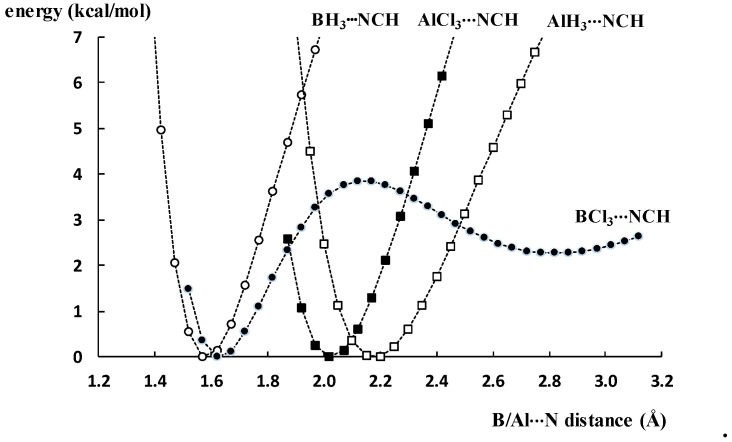
The potential energy curves for the following complexes: BCl_3_⋅⋅⋅NCH (full circles), BH_3_⋅⋅⋅NCH (open circles), ACl_3_⋅⋅⋅NCH (full squares), and AlH_3_⋅⋅⋅NCH (open squares).

Potential energy curves were constructed for all complexes analysed here, and the double minimum was found only in one case, i.e., for the BCl_3_⋅⋅⋅NCH complex. This curve and other potential energy curves for the three additional complexes are presented in Figure 3. Because of differences in the scale for each complex considered, the curves for BH_3_⋅⋅⋅NCH, AlCl_3_⋅⋅⋅NCH, and AlH_3_⋅⋅⋅NCH complexes are restricted only to the proximity of energetic minima. Each of the curve is “normalised” separately. This means that, for each complex, the differences between their energies and the energetic minimum are plotted versus the corresponding Al/B⋅⋅⋅N distances.

It seems that the existence of two potential energy minima for the BCl_3_⋅⋅⋅NCH complex results from the influence of chlorine substituents that obscure the boron centre. One may consider that the interaction between the BCl_3_ and NCH units contains the electrostatic interactions between the nitrogen centre with the boron centre and with the chlorine substituents. The first N⋅⋅⋅B interaction is attractive, while the N⋅⋅⋅Cl interactions are repulsive. This is the electrostatic balance mentioned above here. In contrast, the chlorine substituents cannot obscure the aluminium centre that is characterised by a greater volume than the boron one. However, it seems that the transfer from one configuration characterised by the shallow minimum into the configuration corresponding to the deeper one in the BCl_3_⋅⋅⋅NCH complex may be easily forced, for example, by crystal structure forces, since the potential barrier height for such a transfer amounts to 1.6 kcal/mol only.

**Figure 4:**
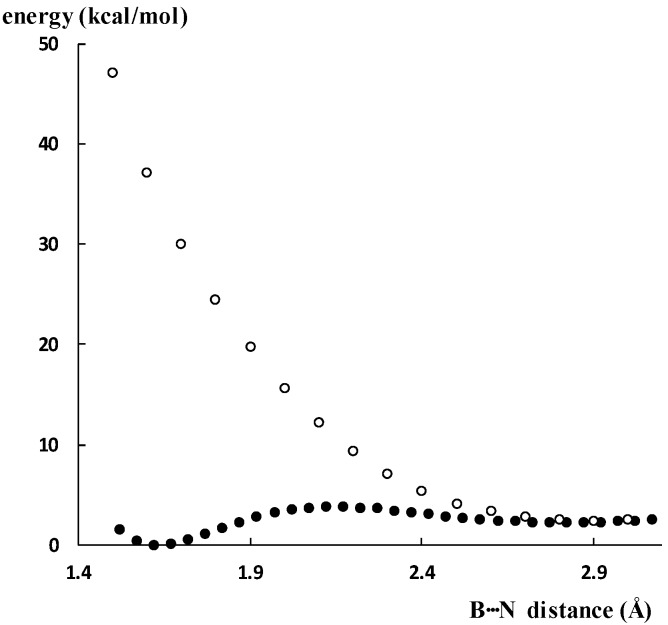
The potential energy curves for the relaxed BCl_3_⋅⋅⋅NCH complex (full circles) and for the BCl_3_⋅⋅⋅NCH complex with rigid Lewis acid and Lewis base units (open circles).

Figure 4 presents the potential energy curve only for the BCl_3_⋅⋅⋅NCH complex. However, the potential energy curve in Figure 4 is more flattened than the corresponding one in Figure 3 because of scale reasons. Another curve presented in Figure 4 also presents energies of this complex for various B⋅⋅⋅N distances; however, the rigid HCN and BCl_3_ moieties are considered as possessing geometries corresponding to the isolated species in their energetic minima. This means that these geometries do not change for different B⋅⋅⋅N distances. This curve is normalised according to the global energy minimum of the complex. One can recall that, in the case of the curve with two minima for each B⋅⋅⋅N distance, the other geometrical parameters are relaxed (optimised). One can see (Figure 4) that, for the rigid interacting units, a monotonic increase of energy is observed with the shortening of the B⋅⋅⋅N distance. This means that the increase of repulsion between chlorine centres and nitrogen is not compensated for by the boron⋅⋅⋅nitrogen attraction. In the case of “relaxed” geometries, the α-angle increases with the shortening of the B⋅⋅⋅N distance. The latter results in weaker Cl⋅⋅⋅N repulsions than for “the rigid case”. These repulsions for relaxed geometries are compensated for by the B⋅⋅⋅N attraction, resulting in the occurrence of the second deeper potential energy well for the B⋅⋅⋅N distance equal to 1.628 Å (see Table 2). However, other interaction energy terms should also be taken into account. The Pauli repulsion is weaker for relaxed geometries than for the rigid interacting species, whereas the orbital energy related to the electron charge shifts is also more important for shorter B⋅⋅⋅N distances.

### 2.3. Covalency of Triel Bonds

The former results discussed here show that the majority of triel bonds possess characteristics of covalent bonds. This is in agreement with the experimental data since the X-ray and neutron diffraction results concerning crystal structures indicate that coordination four occurs most often for the triel centres [24]; in other words, the trivalent triel centres are very reactive and they interact strongly with nucleophiles.

Quantum Theory of Atoms in Molecules (QTAIM) [39,40] calculations were performed here at the level corresponding to the optimised geometries, MP2/aug-cc-pVTZ - the second-order Møller–Plesset perturbation theory (MP2) calculations with the aug-cc-pVTZ basis set. Table 3 shows the characteristics of Al/B⋅⋅⋅N/Cl bond critical points (BCPs) for links between the trivalent triel units and the Lewis base species. The electron density at the corresponding BCP, ρ_BCP_, is situated between 0.006 au for the BCl_3_⋅⋅⋅N_2_ complex and 0.131 au for the BCl_3_⋅⋅⋅NH_3_ complex. The first value is typical for weak van der Waals interactions, while the latter one is typical for covalent bonds that are characterised by values of ~0.1 au [39,40]. Few complexes analysed in this study possess ρ_BCP_ values outweighing 0.1 au.

The typical QTAIM characteristic of the covalent character of the atom–atom link is the negative value of the Laplacian of electron density at the corresponding BCP, ∇^2^ρ_BCP_. This negative value that indicates the concentration of the electron density in the interatomic region is observed only for the BCl_4_^−^ anion. However, it is often assumed that, even for positive ∇^2^ρ_BCP_ values, the negative value of the total electron energy density at the BCP, H_BCP_, confirms the partly covalent character of interaction [41,42,43]. The majority of the links presented in Table 3 are characterised by the negative H_BCP_ values. The positive H_BCP_ values correspond to the weakest interactions. However, there is no correlation between binding or interaction energy and the QTAIM characteristics: ρ_BCP_, ∇^2^ρ_BCP_, or H_BCP_. For example, the linear correlation coefficient for the dependence between the ρ_BCP_ and interaction energy, E_int_, is equal to 0.517. The ρ_BCP_ parameter is related to the part of the interaction connected with its covalent character and not to the total interaction energy [44]. It seems that, for the part of complexes analysed here, the electrostatic interaction is more important than the term related to covalency—the orbital energy. For example, for the BCl_3_⋅⋅⋅NH_3_ complex mentioned above, the ρ_BCP_ value amounts to 0.131 au, while, for the AlCl_3_⋅⋅⋅NH_3_ complex, it is equal to 0.060 au (Table 3). The interaction and binding energies for these systems are very close to each other (Table 1). However, the electron density at the BCP concerns the covalent character of interaction that is greater for the boron system than for the aluminium one.

**Figure 5:**
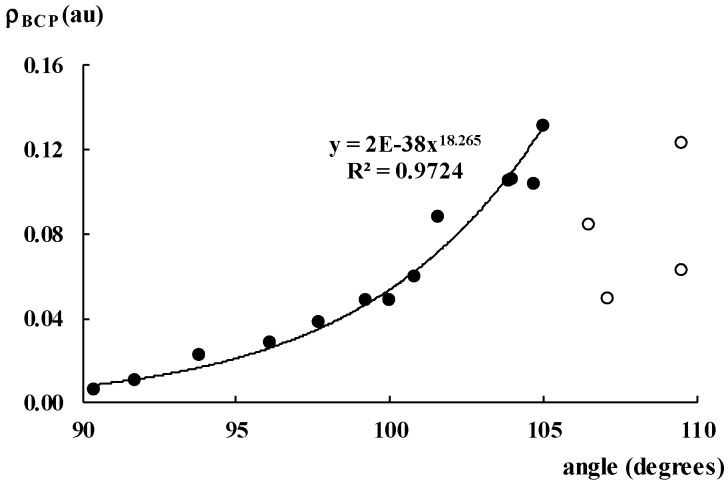
The exponential relationship between the α-angle (degrees, Scheme 1) and the electron at the B/Al⋅⋅⋅N BCP (au), ρ_BCP_; the B/Al⋅⋅⋅Cl contacts of anionic species (open circles) are excluded from this dependence.

The α-parameter presented earlier here (Scheme 1) also expresses the covalent character of the interaction since it informs about the transformation from the triel planar trivalent system into the tetrahedral structure. Figure 5 shows the excellent exponential correlation between α and ρ_BCP_. The anionic systems significantly differing from the remaining complexes are excluded from this dependence; they are presented in this figure only for comparison. The results of the decomposition of interaction energies are shown in Table 4. The decomposition was performed for the density functional theory (DFT) calculations. However, there is an excellent agreement between the DFT and MP2/aug-cc-pVTZ results; for example, a linear correlation between DFT and MP2 interaction energies is observed, *R^2^* = 0.981. Thus, the results of decomposition are well fitted to the MP2 results of calculations discussed here.

Let us return to the BCl_3_⋅⋅⋅NH_3_ and AlCl_3_⋅⋅⋅NH_3_ pair of complexes discussed above here. They are characterised by a similar strength of interaction; however, for the former one, the ρ_BCP_ is over two times greater than for the latter complex. A negative H_BCP_ is observed for the former complex and it is also negative for the latter one, but the latter value is very close to zero. This may suggest that, for the boron moiety, the B⋅⋅⋅N contact is covalent in nature, at least partly, while the Al⋅⋅⋅N link for the latter complex is electrostatic in nature. This also supports the suggestion of Gillespie and Popelier [44] that electron density at the bond critical point expresses the covalent nature of interaction. Furthermore, for the aluminium complex, the electrostatic interaction energy term is almost two times greater than the orbital energy, which concerns the absolute values (Table 4). In the case of the BCl_3_⋅⋅⋅NH_3_ complex, another situation is observed, where orbital and electrostatic energies are comparable to each other. The results of Table 4 show that there is no specific difference between boron and aluminium complexes or between the triel centres connected with chlorine or hydrogen substituents. One can also see that the dispersion energy is much less important with respect to stabilising the systems considered than the electrostatic and orbital terms; this is observed for all complexes analysed.

**Figure 6:**
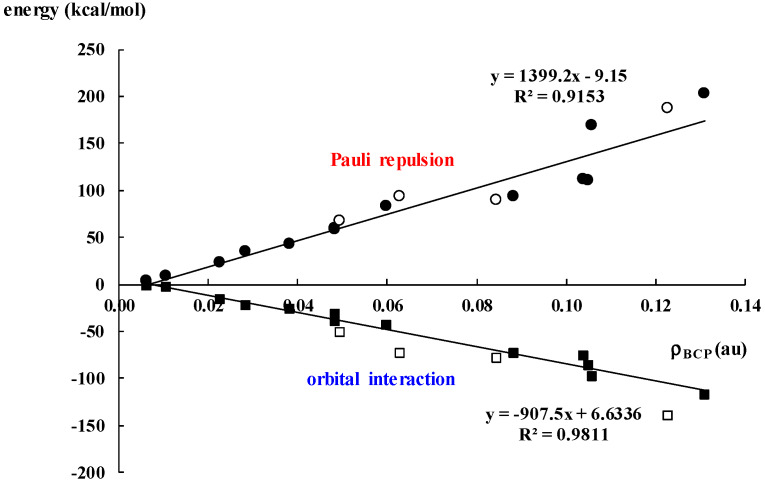
The linear correlations between the electron density at the B/Al⋅⋅⋅N/Cl BCP (au), ρ_BCP_, and the interaction energy terms, Pauli repulsion (circles) and orbital energy (squares); the anionic species are designated by open circles and open squares.

Figure 6 presents linear correlations between the electron density at the B/Al⋅⋅⋅N/Cl BCP, ρ_BCP_, and two terms of the interaction energy resulting from its partitioning: Pauli repulsion (ΔE_Pauli_) and orbital energy (ΔE_orb_) (see *Computational Details* section). These are good correlations despite them concerning the sample of complexes containing various kinds of interactions. These two terms, ΔE_Pauli_ and ΔE_orb_, are often related to the covalent character of interaction [43], especially the orbital energy, which is connected with the electron charge shifts resulting from complexation. It was pointed out in several studies that the dominance of the ΔE_orb_ term testifies to the covalent character of interaction [43]. It is worth mentioning that ρ_BCP_ does not correlate with ΔE_elstat_, with the ΔE_disp_ term, or with the total interaction energy, as mentioned above.

## 3. Conclusions

The simple complexes of boron and aluminium trichlorides and trihydrides with Lewis base units such as hydrogen cyanide, molecular nitrogen, ammonia, and chloride anion were analysed. These complexes are linked through interactions that were named in recent studies as triel bonds. The majority of interactions analysed here are classified as strong ones that possess characteristics of covalent bonds. For the corresponding complexes of the latter interactions, the complexation leads to the transformation of the planar trigonal trichlorides and trihydrides into structures containing a tetrahedral triel centre (boron or aluminium). In particular, such a situation occurs for complexes of the chloride anion, while perfect tetrahedron structures are observed for BCl_4_^−^ and AlCl_4_^−^ species.

In a few cases, weak triel bonds are observed, especially for the BCl_3_⋅⋅⋅N_2_ complex and one of configurations of the BCl_3_⋅⋅⋅NCH complex. The interactions linking the AlCl_3_⋅⋅⋅N_2_ and AlH_3_⋅⋅⋅N_2_ complexes may also be classified as weak ones. For these weak interactions, the Lewis acid unit, BCl_3_, AlCl_3_, or AlH3, retains almost a flat structure in the complex.

Different parameters may describe the character of triel bonds. Among numerous findings presented in this study, it is worth recalling that the angle parameter, α, describes the transformation from the trigonal planar structure into the tetrahedron; this parameter corresponds to the strength of interaction. It is discussed here that the electron density at the BCP corresponds rather to the part of the energy of interaction that is related to the electron charge shifts, and not to the total interaction energy.

## 4. Materials and Methods

### Computational Details

The calculations were carried out with the use of Gaussian16 set of codes [45]. These are the second-order Møller–Plesset perturbation theory (MP2) [46] calculations with the aug-cc-pVTZ basis set [47]. Frequency calculations were performed at the same MP2/aug-cc-pVTZ level to confirm that the optimised structures of complexes correspond to the energetic minima.

The Quantum Theory of Atoms in Molecules (QTAIM) [39,40] was also used to analyse bond critical points (BCPs) of the intermolecular B/Al⋅⋅⋅N and B/Al⋅⋅⋅Cl contacts. The AIMAll program [48] was applied to carry out QTAIM calculations, as well as to calculate the electrostatic potentials (EPs). The analysis of the electron charge density shifts, being the result of complexation, was performed with the use of the Natural Bond Orbital (NBO) method [33,34]. The NBO calculations were performed at the BP86-D3/TZ2P level; i.e., with the use of the BP86 functional [49,50] in conjunction with the Grimme dispersion corrections (BP86-D3) [51] and the uncontracted Slater-type orbitals (STOs) as basis functions with triple-ζ quality for all elements [52]. The same BP86-D3/TZ2P level was applied to perform decomposition energy calculations. The NBO [53] and decomposition energy [54,55] calculations were carried out with the use of the ADF2017 program package [55,56] and using geometries of complexes optimised previously at the MP2/aug-cc-pVTZ level. The total interaction energy for the ADF partitioning is composed according to equation given below.

ΔE_int_ = ΔE_elstat_ + ΔE_Pauli_ + ΔE_orb_ + ΔE_disp_. (1)

The term ΔE_elstat_ corresponds to the quasi-classical electrostatic interaction between the unperturbed charge distributions of atoms; it is usually attractive (negative). The Pauli repulsion, ΔE_Pauli_, is the energy change associated with the transformation from the superposition of the unperturbed electron densities of the isolated fragments to the wave function that properly obeys the Pauli principle through antisymmetrisation and renormalisation of the product wave function. This repulsive term (positive) comprises the destabilising interactions between electrons of the same spin on either fragment. The orbital interaction, ΔE_orb_, corresponds to the charge transfer and polarisation effects.

## Figures and Tables

**Table 1:** The energetic characteristics of complexes analysed (in kcal/mol), the interaction and binding energy (E_int_ and E_bin_, respectively), the deformation energy (E_def_), and the basis set superposition error (BSSE); a and b superscripts stand for complexes linked by weak and strong triel bonds, respectively.

Complex	E_bin_	E_int_	BSSE	E_def_
BCl_3_⋅⋅⋅NCH ^a^	−3.2	−3.5	0.8	0.3
BCl_3_⋅⋅⋅NCH ^b^	−3.8	−23.6	2.5	19.8
BCl_3_⋅⋅⋅N_2_^a^	−1.5	−1.5	0.6	0.0
BCl_3_⋅⋅⋅NH_3_^b^	−26.7	−50.4	2.7	23.6
BCl_4_^− b^	−45.6	−84.9	2.8	39.2
BH_3_⋅⋅⋅NCH ^b^	−18.6	−30.4	1.2	11.8
BH_3_⋅⋅⋅N_2_^b^	−6.1	−14.2	1.3	8.1
BH_3_⋅⋅⋅NH_3_^b^	−30.8	−44.0	1.1	13.2
BH_3_Cl^− b^	−34.1	−50.9	1.7	16.8
AlCl_3_⋅⋅⋅NCH ^b^	−23.8	−29.5	1.5	5.7
AlCl_3_⋅⋅⋅N_2_^a^	−7.0	−9.0	1.3	2.0
AlCl_3_⋅⋅⋅NH_3_^b^	−38.8	−45.7	1.6	6.9
AlCl_4_^− b^	−76.0	−98.5	1.7	22.5
AlH_3_⋅⋅⋅NCH ^b^	−17.7	−20.6	0.7	2.9
AlH_3_⋅⋅⋅N_2_^a^	−5.8	−6.5	0.7	0.7
AlH_3_⋅⋅⋅NH_3_^b^	−28.7	−32.8	0.6	4.1
AlH_3_Cl^− b^	−52.2	−66.6	1.1	14.4

**Table 2:** The geometrical and the Natural Bond Orbital (NBO) parameters; B/Al⋅⋅⋅N/Cl is the distance between the triel centre and the centre of the Lewis base unit (in Å), α-angle (in degrees) is defined in Scheme 1, Ch-shift is the electron charge shift from the Lewis base unit to the Lewis acid that results from complexation (au), while B/Al is the charge (in au) of the triel centre in the complex,^1^ and Pol-B/Al is the percentage of the electron density of the B/Al⋅⋅⋅N/Cl bond orbital at the triel centre; a and b superscripts stand for complexes linked by weak and strong triel bonds, respectively.

Complex	B/Al⋅⋅⋅N/Cl	α-angle	Ch-shift	B/Al	Pol-B/Al
BCl_3_⋅⋅⋅NCH ^a^	2.817	91.7	−0.012	0.344	-
BCl_3_⋅⋅⋅NCH ^b^	1.628	104.0	−0.292	0.265	22.1
BCl_3_⋅⋅⋅N_2_^a^	3.083	90.4	−0.005	0.320	-
BCl_3_⋅⋅⋅NH_3_^b^	1.614	105.0	−0.411	0.312	22.8
BCl_4_^− b^	1.862	109.5	−0.690	0.238	30.4
BH_3_⋅⋅⋅NCH ^b^	1.582	103.9	−0.260	−0.275	77.5
BH_3_⋅⋅⋅N_2_^b^	1.622	101.6	−0.226	−0.287	21.1
BH_3_⋅⋅⋅NH_3_^b^	1.652	104.7	−0.396	−0.234	79.4
BH_3_Cl^− b^	1.968	106.5	−0.549	−0.289	74.5
AlCl_3_⋅⋅⋅NCH ^b^	2.024	100.0	−0.126	1.327	90.9
AlCl_3_⋅⋅⋅N_2_^a^	2.213	96.1	−0.099	1.297	92.5
AlCl_3_⋅⋅⋅NH_3_^b^	1.999	100.8	−0.186	1.325	90.4
AlCl_4_^− b^	2.164	109.5	−0.440	1.240	83.5
AlH_3_⋅⋅⋅NCH ^b^	2.099	97.7	−0.107	0.903	91.5
AlH_3_⋅⋅⋅N_2_^a^	2.290	93.8	−0.114	0.916	93.5
AlH_3_⋅⋅⋅NH_3_^b^	2.071	99.2	−0.179	0.899	8.9
AlH_3_Cl^− b^	2.255	107.1	−0.376	0.822	15.1

^1^ Charges of triel centres in isolated Lewis acid units: BCl_3_ + 0.312 au, BH_3_ + 0.299 au, AlCl_3_ + 1.400 au, AlH_3_ + 1.125 au.

**Table 3:** The Quantum Theory of Atoms in Molecules (QTAIM) parameters (in au), the electron density at the bond critical point (BCP) (ρ_BCP_), the Laplacian of the electron density (∇^2^ρ_BCP_), and the total electron energy density at BCP (H_BCP_); a and b superscripts stand for complexes linked by weak and strong triel bonds, respectively.

Complex	ρ_BCP_	∇^2^ρ_BCP_	H_BCP_
BCl_3_⋅⋅⋅NCH ^a^	0.011	0.035	0.001
BCl_3_⋅⋅⋅NCH ^b^	0.106	0.403	−0.070
BCl_3_⋅⋅⋅N_2_^a^	0.006	0.022	0.001
BCl_3_⋅⋅⋅NH_3_^b^	0.131	0.256	−0.111
BCl_4_^− b^	0.123	−0.055	−0.116
BH_3_⋅⋅⋅NCH ^b^	0.105	0.662	−0.056
BH_3_⋅⋅⋅N_2_^b^	0.088	0.652	−0.038
BH_3_⋅⋅⋅NH_3_^b^	0.104	0.444	−0.069
BH_3_Cl^− b^	0.084	0.132	−0.065
AlCl_3_⋅⋅⋅NCH ^b^	0.048	0.310	0.002
AlCl_3_⋅⋅⋅N_2_^a^	0.028	0.164	0.003
AlCl_3_⋅⋅⋅NH_3_^b^	0.060	0.340	−0.005
AlCl_4_^− b^	0.063	0.292	−0.011
AlH_3_⋅⋅⋅NCH ^b^	0.038	0.244	0.004
AlH_3_⋅⋅⋅N_2_^a^	0.023	0.125	0.003
AlH_3_⋅⋅⋅NH_3_^b^	0.048	0.274	0.000
AlH_3_Cl^− b^	0.050	0.226	−0.006

**Table 4:** The interaction energy terms (in kcal/mol) for systems analysed here (see *Computational Details* section for description and designations); a and b superscripts stand for complexes linked by weak and strong triel bonds, respectively.

Complex	ΔE_int_	ΔE_Pauli_	ΔE_elstat_	ΔE_orb_	ΔE_disp_
BCl_3_⋅⋅⋅NCH ^a^	−1.9	8.5	−5.8	−2.6	−2.1
BCl_3_⋅⋅⋅NCH ^b^	−18.5	168.9	−86.9	−98.7	−1.9
BCl_3_⋅⋅⋅N_2_^a^	−1.0	3.7	−1.7	−1.3	−1.7
BCl_3_⋅⋅⋅NH_3_^b^	−43.8	202.8	−125.8	−117.7	−3.1
BCl_4_^− b^	−80.9	186.8	−126.6	−140.2	−0.9
BH_3_⋅⋅⋅NCH ^b^	−36.7	109.9	−59.3	−86.2	−1.1
BH_3_⋅⋅⋅N_2_^b^	−23.6	93.6	−42.3	−73.8	−1.1
BH_3_⋅⋅⋅NH_3_^b^	−45.9	110.7	−78.1	−76.7	−1.9
BH_3_Cl^− b^	−53.8	88.9	−63.1	−78.8	−0.9
AlCl_3_⋅⋅⋅NCH ^b^	−32.7	57.4	−47.6	−40.0	−2.6
AlCl_3_⋅⋅⋅N_2_^a^	−10.1	35.2	−20.5	−22.4	−2.3
AlCl_3_⋅⋅⋅NH_3_^b^	−43.7	82.1	−78.7	−43.6	−3.6
AlCl_4_^− b^	−95.1	93.6	−113.2	−73.8	−1.6
AlH_3_⋅⋅⋅NCH ^b^	−21.0	41.9	−34.3	−27.2	−1.5
AlH_3_⋅⋅⋅N_2_^a^	−8.5	22.3	−13.7	−16.0	−1.2
AlH_3_⋅⋅⋅NH_3_^b^	−32.7	59.5	−58.6	−31.2	−2.3
AlH_3_Cl^− b^	−65.5	67.1	−81.2	−50.8	−0.7
